# *Rhabditophanes diutinus* a parthenogenetic clade IV nematode with dauer larvae

**DOI:** 10.1371/journal.ppat.1009113

**Published:** 2020-12-03

**Authors:** Alex Dulovic, Tess Renahan, Waltraud Röseler, Christian Rödelsperger, Ann M. Rose, Adrian Streit

**Affiliations:** 1 Department of Integrative Evolutionary Biology, Max Planck Institute for Developmental Biology, Tübingen, Baden-Württemberg, Germany; 2 Department of Medical Genetics, University of British Columbia, Vancouver, British Columbia, Canada; La Trobe University, AUSTRALIA

## Abstract

Comparative studies using non-parasitic model species such as *Caenorhabditis elegans*, have been very helpful in investigating the basic biology and evolution of parasitic nematodes. However, as phylogenetic distance increases, these comparisons become more difficult, particularly when outside of the nematode clade to which *C*. *elegans* belongs (V). One of the reasons *C*. *elegans* has nevertheless been used for these comparisons, is that closely related well characterized free-living species that can serve as models for parasites of interest are frequently not available. The Clade IV parasitic nematodes *Strongyloides* are of great research interest due to their life cycle and other unique biological features, as well as their medical and veterinary importance. *Rhabditophanes*, a closely related free-living genus, forms part of the Strongyloidoidea nematode superfamily. *Rhabditophanes diutinus* (= *R*. sp. KR3021) was included in the recent comparative genomic analysis of the Strongyloididae, providing some insight into the genomic nature of parasitism. However, very little is known about this species, limiting its usefulness as a research model. Here we provide a species description, name the species as *R*. *diutinus* and investigate its life cycle and subsequently gene expression in multiple life stages. We identified two previously unreported starvation induced life stages: dauer larvae and arrested J2 (J2A) larvae. The dauer larvae are morphologically similar to and are the same developmental stage as dauers in *C*. *elegans* and infective larvae in *Strongyloides*. As in *C*. *elegans* and *Strongyloides*, dauer formation is inhibited by treatment with dafachronic acid, indicating some genetic control mechanisms are conserved. Similarly, the expression patterns of putative dauer/infective larva control genes resemble each other, in particular between *R*. *diutinus* and *Strongyloides* spp. These findings illustrate and increase the usefulness of *R*. *diutinus* as a non-parasitic, easy to work with model species for the Strongyloididae for studying the evolution of parasitism as well as many aspects of the biology of *Strongyloides* spp, in particular the formation of infective larvae.

## Author summary

Parasitic worms are an issue of great medical, veterinary, agricultural and economic importance, yet little is known about how worms become parasites. Comparative studies with non-parasitic model species like *C*. *elegans* have been useful, however, this usefulness decreases as the evolutionary distance between the species increases. One way to combat this is by having more well-studied closely related species to parasites of interest. To address this, we provide information about *Rhabditophanes diutinus*, a free-living nematode that is part of the same superfamily as the medically and veterinary important *Strongyloides* parasites. We provide analysis on its life cycle, in particular on two starvation induced life stages, along with gene expression data. Overall, this important information illustrates and improves the use of *R*. *diutinus*, as a non-parasitic model species for studying parasite evolution and basic biology within *Strongyloides*.

## Introduction

Parasitic nematodes are of great medical, agricultural, veterinary and economic importance, yet their study and control has been often neglected [1]. As a result, multiple parasitic nematodes are causative agents of Neglected Tropical Diseases (NTDs) [2] and human parasitic nematodes alone cause annually a loss of over 5 million disability adjusted life years [3]. While parasitism is generally ubiquitous [4], it is known to have evolved independently within nematodes on upto 18 separate occasions [5], meaning it can only be studied at specific defined transitions. Nematodes themselves consist of 5 major clades [6], all of which contain parasites, yet there is still a lack of understanding of how parasitism has arisen. Since each transition to parasitism was an independent historical event, each transition must therefore be studied independently. To what extent some or all of these separate evolutionary transitions followed common general principles is unclear.

The so-called dauer hypothesis for nematode parasitism [7] was proposed as one such common principle that might have been involved in multiple transitions to parasitism. This hypothesis states that dauer larvae, which exist in a number of non-parasitic nematodes, served as a pre-adaptation to and eventually evolved into infective larvae. Dauer larvae themselves are a specialized long lasting life stage produced in response to extreme environmental conditions, allowing the population to survive [8]. Their formation has been well-studied in the model nematode *C*. *elegans* and for an overview of the genetic pathways involved in their formation and dauers themselves, we encourage the reader to consult [7–10].

Recent attempts to understand the biology and the evolution of parasitic nematodes through comparative studies between parasitic species and *C*. *elegans*, have begun to identify conserved canonical dauer signaling pathways involved in the formation of infective larvae, providing support to the dauer hypothesis [11,12]. In particular, the functions of the nuclear hormone receptor DAF-12 and its ligand dafachronic acid which are the most downstream effectors of dauer development appear highly conserved across nematodes [13–20], with suppression of DAF-12 resulting in a loss of dauer or infective development or a redirection towards free-living stages. However, clear differences such as the absence of key dauer control genes in parasitic species [21] or changes in functions or logic of conserved genes and their interactions [10,22], means substantial further analysis is required. The use of *C*. *elegans* in comparative studies is often a problem, as while it has a substantial knowledge base, it is often too evolutionarily remote from the studied parasitic species, resulting in phylogenetic separation being the likeliest cause of any observed differences [5,6]. Therefore, it is through studying closely related free-living and parasitic species, that the biology and the evolution of parasitic nematodes can possibly be better understood. For the clade IV Strongyloididae parasites, who are more divergent from the clade V *C*. *elegans* than humans are from zebrafish [23], this is particularly true.

**Fig 1:**
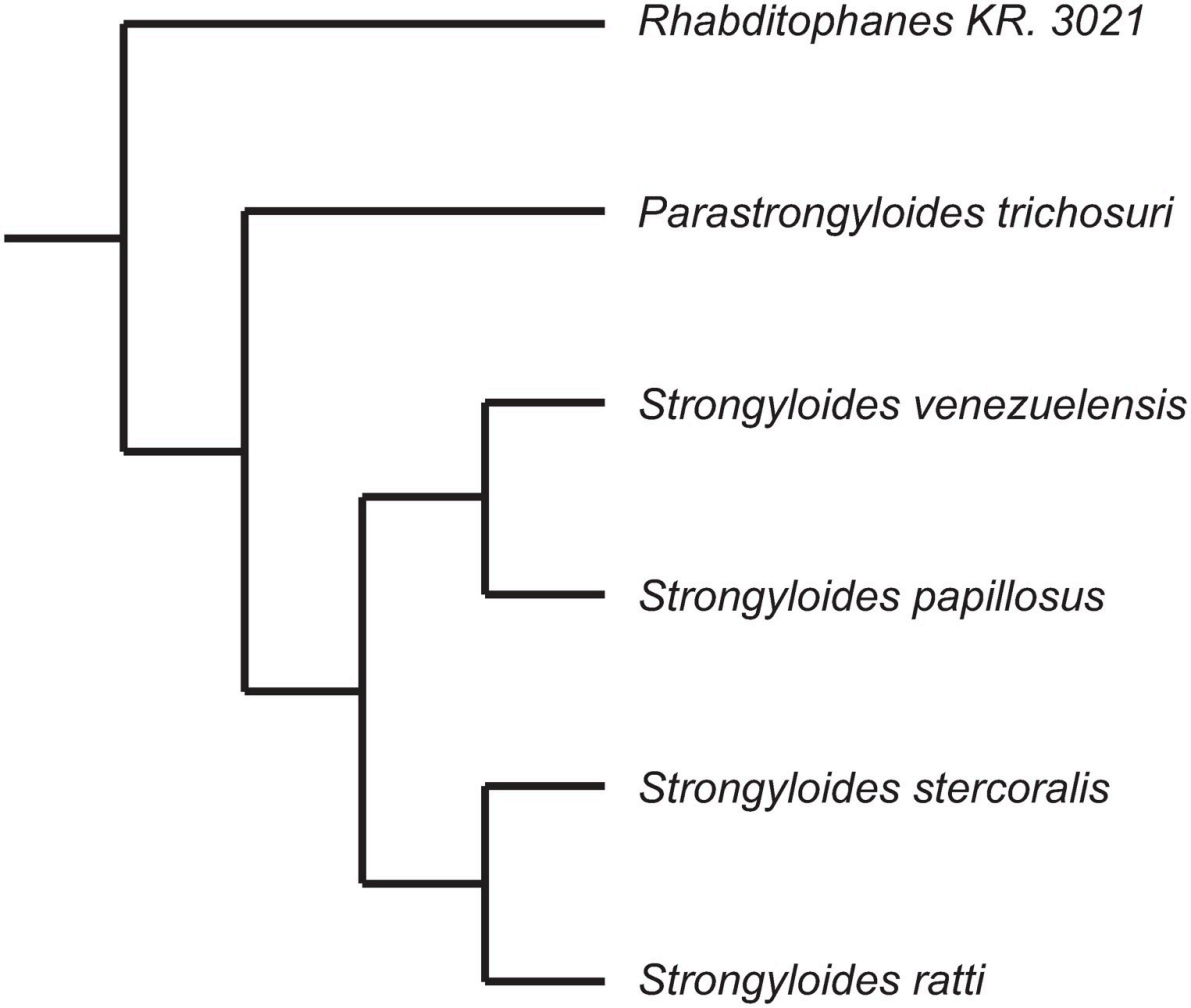
Phylogenetic relationship of *Rhabditophanes* and selected Strongyloididae species based on(5, 25). Only the tree topology is shown. The branch lengths are not informative.

The Strongyloididae together with the Alloinematidae form the Strongyloidoidea superfamily [24], which is a highly attractive model for studying the evolution of parasitism and testing the dauer hypothesis, as it contains the obligate *Strongyloides* parasites, the facultative *Parastrongyloides* parasites and the free-living *Rhabditophanes* nematodes [25]. The phylogeny of these taxa can be found in Fig 1. However, this superfamily is also of great interest in its own right because it contains *Strongyloides stercoralis*, a small intestinal parasite estimated to infect more than 600 million people worldwide [26]. Although frequently asymptomatic, infections can result in deadly disease, particularly in immuno compromised patients [27]. The progeny of host dwelling parasitic *S*. *stercoralis* females can develop to infective third stage larvae (iL3) while still within the host and infect that same host individual (auto-infective cycle). This is in addition to the "normal" life cycle present in all species of *Strongylodies*, whereby these progeny leave the host as young larvae and continue their development into iL3 (direct development), or free-living adults which reproduce sexually (indirect development) [28]. The auto-infective cycle is mostly responsible for severe cases of strongyloidiasis, as it enables low-level infections to persist asymptomatically for many years before self-enhancing [27]. This enhancement results in hyperinfection syndrome and dissemination, which is lethal in most cases if not treated in time [27]. Therefore, investigating the developmental switch between (auto-) infective larvae and free-living worms is important in order to understand the pathogenicity of *S*. *stercoralis*.

As the closest non-parasitic relative of *Strongyloides* spp., *Rhabditophanes* is of particular interest not only with respect to the study of the evolution of parasitism [29] but also as a possible non-parasitic model species to study other aspects of *Stronglyoides* spp. biology, such as the formation of dauer / (auto-) infective larvae. Although it has been used in a small number of comparative studies [25,30–33], relatively little is known about *Rhabditophanes*, as evidenced by the lack of a species name for its most cultured and well-studied member (sp. KR3021).

As *Rhabditophanes* sp. KR3021 fits the niche of being free-living and phylogenetically close to parasites of interest, we aim to provide some much needed basic information of this species. We suggest naming the species *Rhabditophanes diutinus*, in light of both its multiple survival strategies (see below) and the long period of time from its isolation to naming. We provide a formal species description including measurements in the supplement (S1 File). We found that this species can form two long-lived stages in response to starvation, namely a J2 arrested stage (J2A) and dauer larvae, both of which could be recovered with the addition of food. To our knowledge this is the first example of a clade IV nematode that can be induced to enter and exit the dauer stage under laboratory conditions. Its SDS resistance, developmental stage, morphology, longevity, gene expression patterns and inhibition of dauer formation with dafachronic acid strongly suggests that *R*. *diutinus* dauers are homologous to *C*. *elegans* dauers and *Strongyloides* infective larvae. Our results make it highly unlikely that *R*. *diutinus* is secondarily free-living upon reverting from a parasitic life style as had been previously proposed [34], based in part however on the erroneous phylogenetic placement of *Rhabdias bufonis* [35]. Overall, our results illustrate that *R*. *diutinus* is not only an attractive system to study the evolutionary transition to parasitism but also as a non-parasitic model for the study of a group of nematodes that contains important pathogens without the need for host animals.

## Results

### Life Cycle of *Rhabditophanes diutinus*

**Fig 2:**
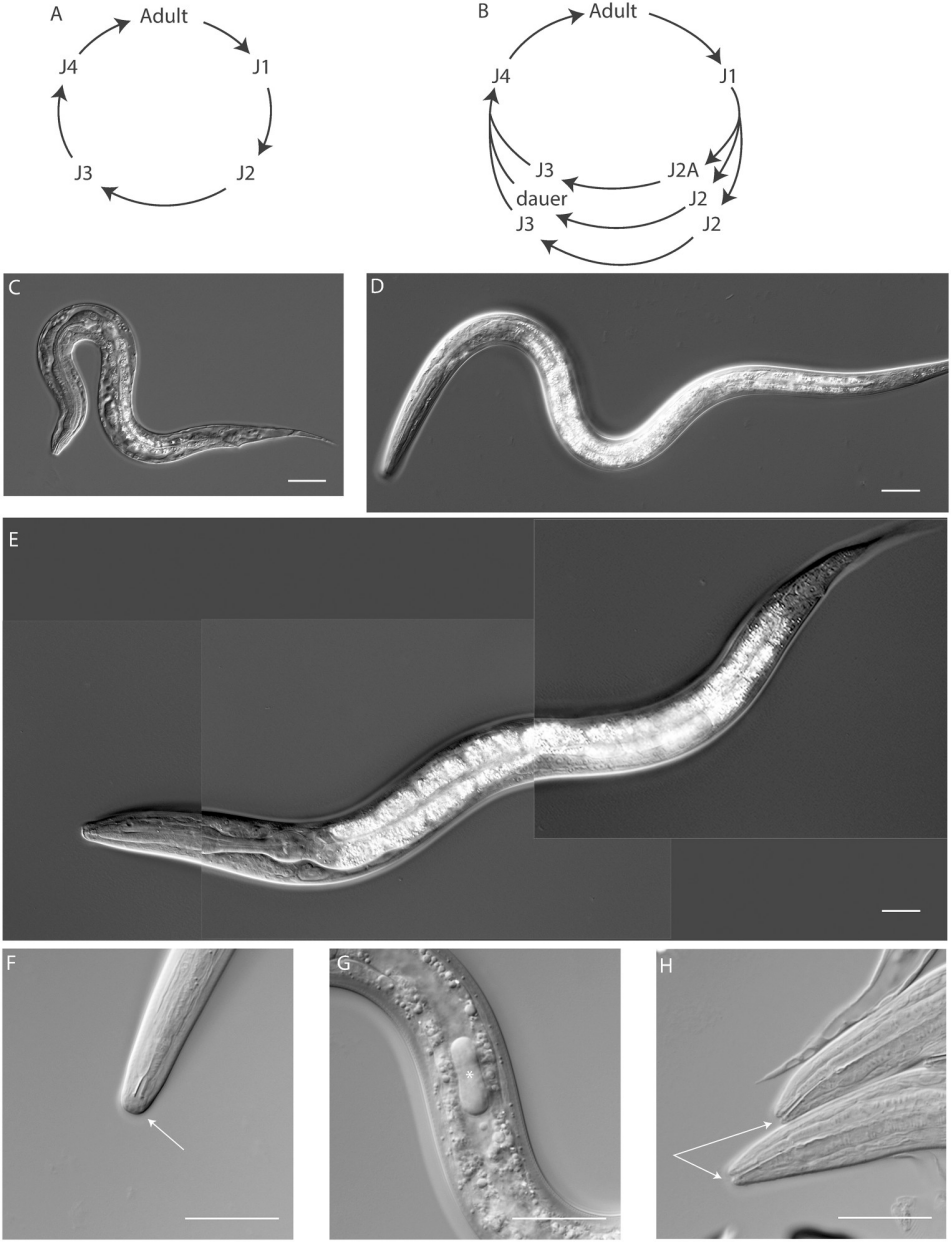
Life cycle and developmental stages of *R*. *diutinus*. (A) previously described simple life cycle according to [25]. (B) Updated life cycle for *R*. *diutinus*. (C) J2A larva. (D) dauer larva. (E) J3 larva for comparison. (F) buccal plug (arrow) and (G) the uppermost of several intestinal plugs (star). (H) Front ends of J2A, lacking a buccal plug but featuring an ordinary narrow open mouth (arrows). All scale bars are 25μm.

Previous descriptions of *R*. *diutinus* (KR3021) state it has a simple free-living cycle consisting of four larval molts from embryos through adults with reproduction occurring by meiotic parthenogenesis [25] (Fig 2A). In other species of *Rhabditophanes*, dauer larvae (identified solely through morphology) in association with arthropods had been previously described, but not in *R*. *diutinus* [36,37]. When examining overgrown *R*. *diutinus* plates, we saw worms of two previously undescribed stages: a dauer-like larvae (Fig 2D) and a small arrested stage larvae (Fig 2C). If we assume that *Rhabditophanes* spp. is secondarily free-living [34], we would expect it to have lost its original dauer stage. As a result, should a functional dauer stage exist, then we would expect this to be newly gained after the reversal to a non-parasitic lifestyle. To determine if *R*. *diutinus* does indeed form dauer larvae that are homologous to the dauer larvae of clade V nematodes, we further investigated the two starvation induced stages. In other nematodes, only dauer larvae which have both a thickened cuticle and buccal plug are able to survive treatment with SDS [38,39]. We found that also in *R*. *diutinus*, only dauer-like larvae were able to survive this treatment and could be recovered on an NGM plate. Examination of these larvae under DIC microscopy, revealed that they contain a 3 part buccal plug, consisting of a plug in the mouth (Fig 2F), a further larger plug located approximately one third of the way down in the intestinal lumen (Fig 2G) and a final series of plugs located throughout the posterior portion of the intestinal lumen.

The arrested small stage larvae (Fig 2C) (from hereon referred to as J2A) were unable to survive SDS treatment. Microscope examination revealed they lack a buccal plug (Fig 2H) or any other dauer specific morphological features and instead appear more similar to J2 larvae, although they have a smaller germline, thickened intestinal lumen and increased lifespan (a full morphological description of adults, J2As, J2s and dauers including measurements can be found in S1 File). For comparative purposes, a J3 worm is included as Fig 2E. Type material will be submitted to the CNRC, the Swedish Museum of Natural History and the State Museum of Natural History in Karlsruhe upon acceptance of the manuscript.

### Dauer and J2A recovery

**Fig 3:**
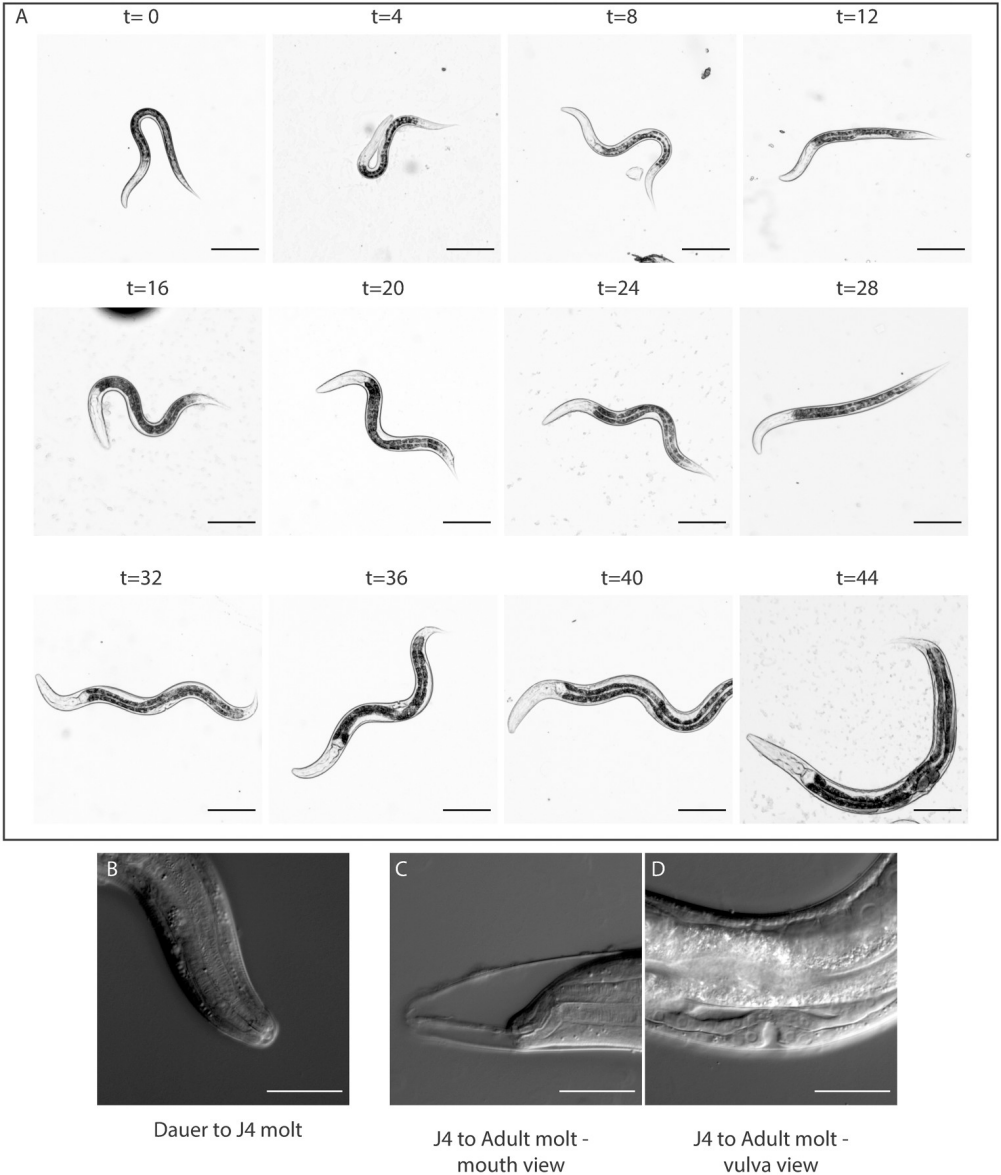
Recovery of dauers into adults in *R*. *diutinus*. (A) Time course of dauer recovery. Images are taken from a time course experiment. Images are shown at 4 hours intervals from dauers (t = 0 hours) through to fully developed reproducing adults (t = 44 hours). (B-D) High magnification DIC images of the two molts, at 16 and 36 hours are shown. Scale bars indicate 100μm. The images shown here are a composite of the experiment and do not constitute a single worm as it develops.

To determine if J2A is a developmental stage of the dauer development route, we transferred J2A larvae onto bacteria-free NGM plates. When recovered without food, J2A larvae were never able to develop into dauer larvae suggesting that they are an alternative to dauer formation. This suggests that *R*. *diutinus* contains multiple strategies for survival when under environmental stress. To confirm that the presumed J2A and dauer larvae were really second and third larval stages respectively, we transferred them onto NGM plates supplemented with *Escherichia coli* OP50 (OP50) bacteria to trigger exit from the enduring stage. Dauer larvae underwent two molts (from dauers into J4 and J4 into adults (Fig 3)), confirming that the dauers are a third stage larvae as in *C*. *elegans* [8]. It took nearly 2 hours for the buccal plug to disappear and a further 4 hours for the intestinal plugs to be removed, perhaps explaining why recovery following SDS treatment took longer than is typical in *P*. *pacificus* or *C*. *elegans*. J2A larvae required 3 molts and nearly 24 hours longer than dauers to become adults. This, in addition to the observation that J2As cannot form dauers confirms that they represent different survival strategies. An updated version of the *R*. *diutinus* life cycle can be seen in Fig 2B. As we have functional dauer larvae within this species, we asked if parts of the regulatory machinery that controls the dauer switch might also be conserved in *R*. *diutinus*.

### Identification of orthologs of known dauer genes in *R*. *diutinus*

We identified orthologs for 53 of the 102 known *C*. *elegans* dauer pathway genes (Table 1)[7,10,11]. Of those present, 45 are one-to-one orthologs. Interestingly, there are striking differences in conservation between different signaling pathways. While the TGF-β (8 of 13) and steroid hormone (12 of 14) signaling pathways are well conserved, much of the insulin signaling pathway is missing (26 of 63 present). As most of these genes were previously shown to be restricted to the genus *Caenorhabditis* based on phylostratigraphic analysis [40], we also identified orthologs of the same genes within all other members of the Strongyloidoidea that have a published genome (Table 2) and found the same genes to be missing across all Strongyloidoidea genomes tested. The full list of genes present in all Strongyloidoidea species tested can be found in S2 File.

**Table 1:** Genes known to be involved in the dauer signaling pathway in *C*. *elegans* that are present within the published *Rhabditophanes diutinus* genome.

Pathway	Total Genes in *C*. *elegans* pathway	1 to 1	1 to Many	Many to 1	Missing	Total Present in *R*. *diutinus*
cGMP	12	6	1	0	5	7
TGF-β	13	8	0	0	5	8
Insulin	63	22	0	4	37	26
Steroid Hormone	14	9	1	2	2	12
**Total**	102	45	2	6	49	53

Genes are grouped according to the pathway in which they belong, and whether they are 1-to-1, 1-to-Many, Many-to-1 orthologs or are missing compared to *C*. *elegans*. An extended version of this table including information on how each ortholog was identified can be found in S2 File.

**Table 2:** Genes present within 5 tested Strongyloididae genomes that are part of the *C*. *elegans* dauer signaling pathway.

Pathway	Orthology	*P*. *trichosuri*	*S*. *stercoralis*	*S*. *ratti*	*S*. *papillosus*	*S*. *venezuelensis*
cGMP	1 to 1	8	7	6	7	7
	1 to Many	0	0	0	0	0
	Many to 1	0	1	1	1	1
	Missing	4	4	5	4	4
	Total present	8	8	7	8	8
TGF-β	1 to 1	11	11	12	12	11
	1 to Many	0	0	0	0	0
	Many to 1	0	0	0	0	0
	Missing	2	2	1	1	2
	Total present	11	11	12	12	11
Insulin	1 to 1	22	22	22	21	22
	1 to Many	0	0	0	0	0
	Many to 1	4	4	4	4	4
	Missing	37	37	37	38	37
	Total present	26	26	26	25	26
Steroid Hormone	1 to 1	9	9	9	9	9
	1 to Many	1	1	1	1	1
	Many to 1	2	2	2	2	2
	Missing	2	2	2	2	2
	Total present	12	12	12	12	12
TOTAL		57	57	57	57	57

Orthologs of known *C*. *elegans* genes were identified through a variety of bioinformatics analyses (see Methods). Genes are grouped according to their pathway, species and what state of orthology they exist in. An extended version of this table with full gene information including the method used to identify each ortholog can be found in S2 File.

### Comparison of the transcriptomes of different *R*. *diutinus* developmental stages

**Fig 4:**
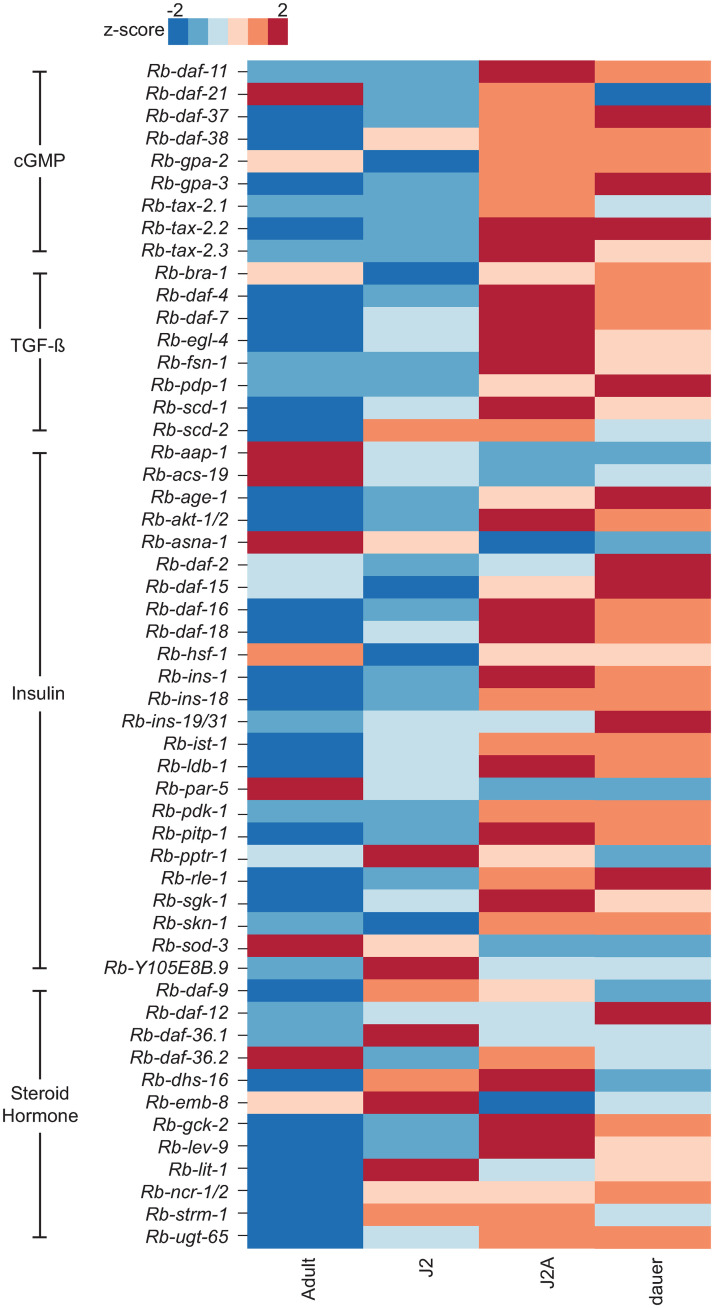
Heatmap of orthologs of known dauer genes in different life stages of *R*. *diutinus*. Transcriptome profile of orthologs of known dauer genes in Adults, J2/J3 larvae, J2As and Dauers. Values shown are mean z score from all 3 biological replicates as replicates were determined to be similar (see S1 Fig). Z score indicates difference in expression between the different stages, with blue indicating a negative z score (-2 to 0) and decreased expression and red indicating a positive z score (0 to 2) and increased expression.

Next, we determined the transcriptional profile of the orthologs of *C*. *elegans* dauer genes in different stages of *R*. *diutinus* (adult, J2/J3, J2A and dauer)(Fig 4). In *C*. *elegans*, these genes are involved in dauer formation but many of them remain expressed in dauers and are also involved in dauer exit while the presence and expression of their orthologs has also been investigated in various parasitic nematodes [7,8,10,11,15,16,41] The majority of these genes are upregulated in one or both of the two long living life stages (41 in J2A, 37 in dauer), with only 7 genes downregulated in both stages. The substantial differences in gene expression combined with the separate PCA grouping of the different stages (S1 Fig), further confirms that J2A and J2 represent different stages, as do J2As and dauers.

In terms of the different dauer development pathways, we found that the cGMP and TGF-β pathways have similar expression in both dauers and J2As, with only *daf-21* being upregulated in J2As but downregulated in dauers. The insulin signaling pathway is substantially differentially expressed across all life stages. Similarly, there are large differences in the steroid hormone pathway including between J2A and dauers. *daf-12*, the most downstream receptor of this pathway, is only upregulated in dauers.

To identify other genes potentially involved in dauer and J2A production or maintenance, we analysed the transcriptomes of J2As, dauers and J2/J3s. Between J2/J3s and J2As, 1371 (10.16%) (Table 3) genes were significantly differentially expressed, further supporting that they are different stages. To identify genes potentially involved in dauer production or maintenance, we compared the transcriptome of dauers and J2/J3s and found that 1409 (10.44%) genes were significantly differentially expressed, with 523 of these being upregulated in dauers. When comparing J2As and dauers, we found only 318 (2.36%) genes were significantly differentially expressed, of which 123 were upregulated only in dauers. Of these, 43 genes were significantly upregulated in dauers compared to both J2A and J2/J3 (S2 File).

**Table 3:** Significantly differentially expressed genes in *R*. *diutinus* between J2/J3s, J2As and dauers.

Stage Comparison	Significantly differentially expressed genes	Non-differentially expressed genes	Upregulated in dauers	Upregulated in J2As	Upregulated in J2/J3s
Dauer vs J2A	318	13178	123	195	
Dauer vs J2/J3	1409	12087	523		886
J2A vs J2/J3	1371	12125		656	715

Total numbers of genes that were significantly expressed between different life stages in *R*. *diutinus*. All genes identified in the transcriptome were compared between different life stages, their fold change determined, and the significance between the two samples. Only those with a log fold change either greater than 2 or lower than -2 and a FDR-corrected p-value of less than 0.05 were determined to be significantly differentially expressed. Dauers were compared with J2/J3 as this represents a similar life stage in terms of molts, and J2As as they are both long living. J2As were compared with J2/J3s as they represent a similar life stage in terms of molts. The complete list of genes for each of these comparisons can be found in S2 File.

### Comparison of the transcriptomes of *R*. *diutinus* dauers and *Strongyloides* infective larvae

**Fig 5:**
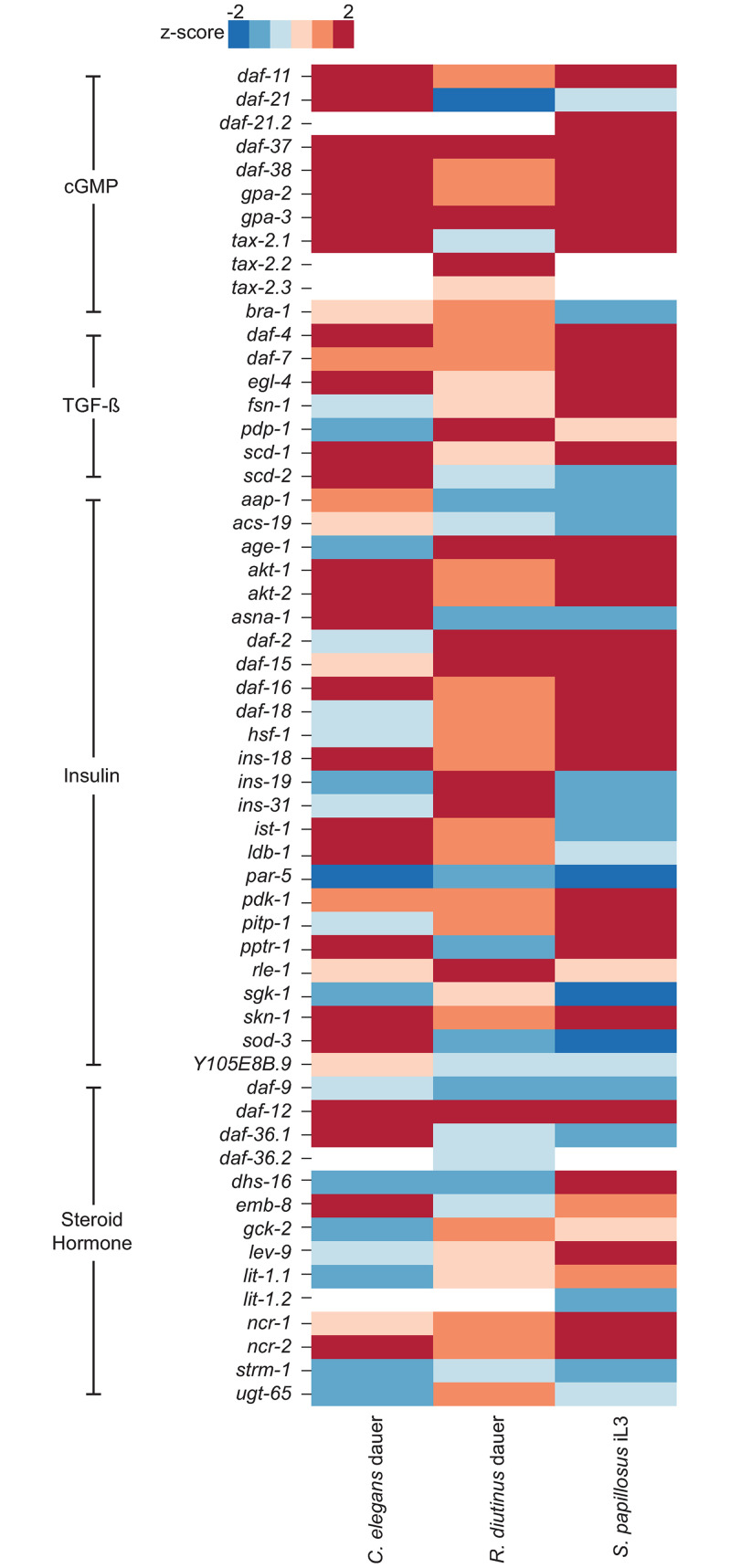
Heatmap of known dauer genes present in all three species (*R*. *diutinus*, *S*. *papillosus* and *C*. *elegans*). Z scores were determined and used to measure differences in expression for known dauer genes in all three species. Only genes that are present in all three species are shown. White indicates that the copy is not present within the genome, blue indicates a negative z score (-2 to 0) and decreased expression and red indicates a positive z score (0 to 2) and increased expression.

To determine whether *R*. *diutinus* dauers might resemble dauer larvae of the phylogenetically remote *C*. *elegans* or infective larvae of the closely-related *S*. *papillosus*, we compared the expression levels of *C*. *elegans* dauer genes for which orthologs are present within all three species (Fig 5). We only studied genes known to be dauer genes in *C*. *elegans* as opposed to global gene expression, as previous research has shown that even between much more phylogenetically close species (*P*. *pacificus and C*. *elegans*), global gene expression is vastly different in dauers [42]. The cGMP pathway appears well conserved in terms of expression profile between all three species, the only difference being *daf-21* which is strongly upregulated in *C*. *elegans* but not in the other two species. The TGF-β pathway is also broadly consistent among the three species, but shows differences in expression of *pdp-1* (upregulated in Strongyloidoidea) and *scd-*2 (upregulated in *C*. *elegans*). The insulin signaling pathway is differentially expressed between all three species. It should be said that large amounts of this pathway are not present within the *R*. *diutinus* or *S*. *papillosus* genomes, however for the genes that are present, there appears to be greater similarity in the profiles of *R*. *diutinus* and *S*. *papillosus* (10 genes up/downregulated in both species only) than *R*. *diutinus* and *C*. *elegans* (3 genes up/downregulated in both species only). Interestingly, *asna-1* which functions as a regulator of insulin secretion [43] is upregulated in *C*. *elegans* but downregulated in *R*. *diutinus* and *S*. *papillosus*. Of the insulin signaling ligands present within *R*. *diutinus* and *S*. *papillosus*, *ins-19/31* is upregulated only in *R*, *diutinus* while *ins-18* is upregulated in all three species. The steroid hormone signaling pathway is differentially expressed among the three species, however *daf-12*, the most downstream gene known to control dauer formation, is strongly upregulated in all three species, suggesting a conserved function within all three species.

### Dafachronic acid prevents dauer formation but does not prevent J2A formation

As the conserved function of *daf-12* in dauer/infective larvae formation has been experimentally demonstrated for *C*. *elegans* and *Strongyloides* spp. [13,15,16], we tested whether the function of *daf-12* is also conserved in *R*. *diutinus*. *R*. *diutinus* larvae were treated with dafachronic acid (DA) or ethanol as a negative control (DA is diluted with ethanol and so all worms were exposed to this solvent) and their developmental stage monitored 14 days later as in [13]. No dauer larvae were seen when plates were supplemented with DA indicating that dauer development was prevented by the DA (Table 4). However, there was no effect on the formation of J2A larvae (95.90% in the negative control compared to 94.40–94.53% when treated with DA) confirming that their development is regulated by a different pathway (Table 4).

**Table 4:** Dafachronic acid treatment prevents the formation of Dauer but not J2A larvae.

	Treatment		
Worm Stage (%)	Ethanol	10μM Δ7 DA	100μM Δ7 DA
Dauers	2.60	0.0 (**)	0.0 (**)
J2A	95.93	94.40	94.53

Young larvae were transferred on new plates supplemented with either Δ7 dafachronic acid (DA) or ethanol (solvent) and incubated for 14 days, after which the amount of dauer and arrested J2 larvae were counted. DA is diluted using Ethanol so all worms were exposed to the same carrier. On each plate, 200 larvae were chosen at random and staged. On plates were no dauer larvae could be counted within the 200, the whole plate was examined to determine if it truly was free from dauer larvae or not. For each treatment, 5 plates were used, and the experiment was repeated three times. Data shown here is a mean of all samples. Mann-Whitney U was performed to determine statistical significance between the different treatments (p = <0.0001 for both).

Taken together the results presented so far, we conclude that *R*. *diutinus* forms dauer larvae which most likely are homologous to dauer larvae of *C*. *elegans* and to iL3s of *Strongyloides* spp.

### Dauer and J2A survival

**Fig 6:**
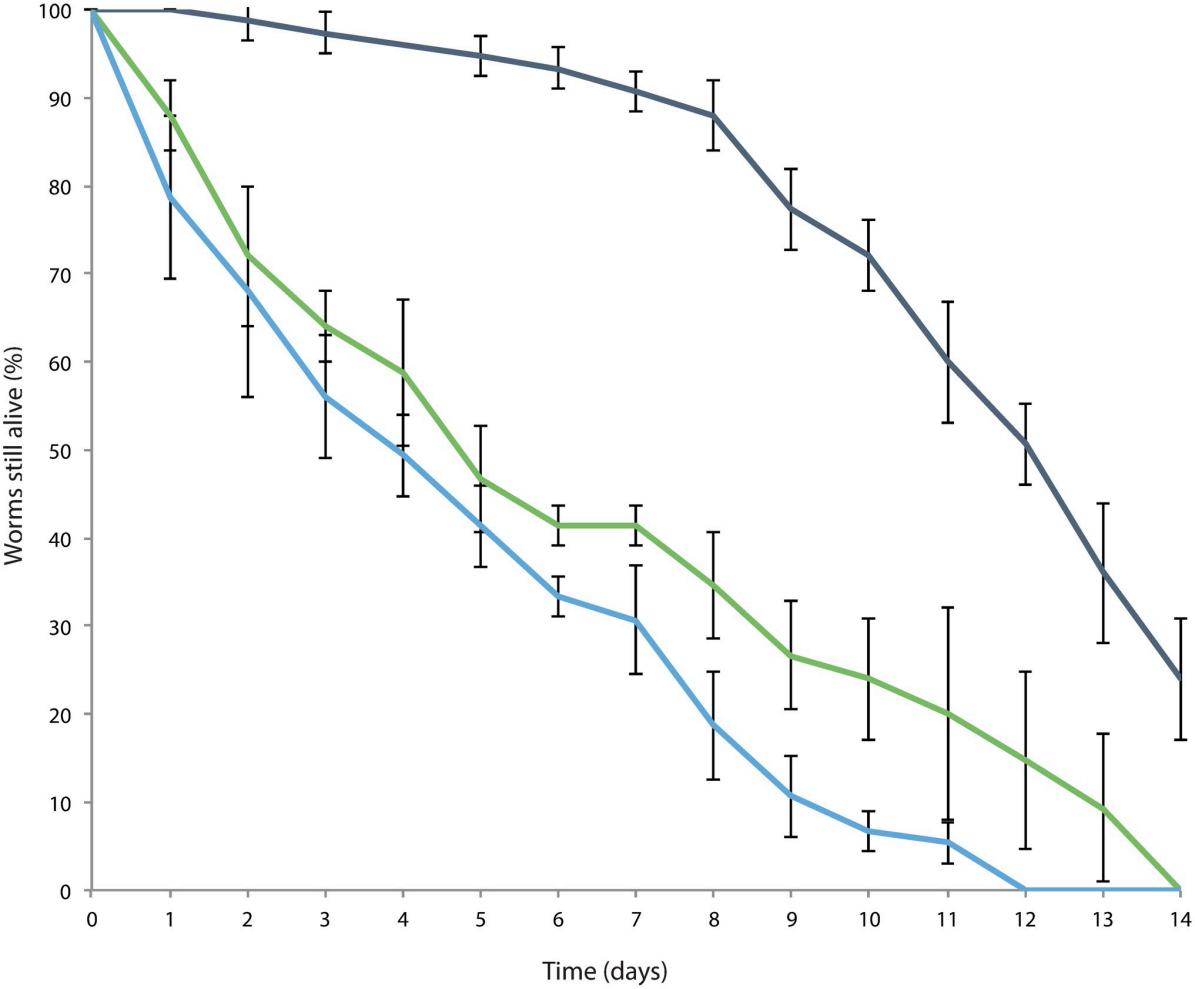
Dauers survive longer than J2A or J2/J3 when on fresh foodless plates. Survival curves of Dauers (dark blue line), J2As (green line) and J2/J3s (light blue line) when transferred onto fresh plates to induce starvation. Per treatment, 75 worms were picked in groups of 5 and checked daily for survival. The experiment was performed at three time points such that each time 25 worms per treatment were analyzed in parallel. The data points shown here are the daily mean of all 75 worms. Error bars are standard deviation.

To determine how long both types of stress induced larvae could survive, dauers and J2A were transferred separately onto bacteria-free NGM plates and observed daily for upto 3 weeks. As a control, normal J2/J3 larvae were also included. As seen in Fig 6, J2/J3 larvae had the shortest lifespan (median 3 days) with all the larvae dead after 12 days. Interestingly, the transfer of these larvae into a foodless environment resulted in them stopping development. The J2A larvae were able to survive significantly longer than the J2 larvae (p-value 0.017) with a median lifespan of 4 days, but all had died after 14 days. As expected, the dauer larvae had a significantly longer lifespan than both the J2 (p-value <0.0001) and J2A larvae (p-value <0.0001) with a median survival of 12 days and 24% were still alive at the end of the 3 weeks. It is currently unknown how long these dauer larvae may be able to last for but they have been seen in plates for upto 3 months.

### The offspring of individuals that developed through dauer are not more likely to form dauers

Next we asked if the progeny of an individual who underwent dauer development are more likely to form dauers themselves. Such an effect could either be caused by genetic differences within the population predisposing some genotypes for dauer development, or it could be due to an epigenetic transgenerational effect. To this end, dauers, J2A and J2/J3 were transferred in groups onto NGM plates supplemented with OP50 and incubated at 15°C. After 2 days of producing offspring, the initial worms were removed from the plates and the total number of offspring produced counted. As seen in Table 5, there was no difference in the number of offspring produced between J2/J3s and recovered dauers and J2A (78.5 for J2/J3, 73.9 for dauer and 73.8 for J2A). After further incubation for 2 weeks, the percentage of dauer larvae present on each plate was then counted. There was no difference in dauer production between J2/J3s (2.95%), recovered J2A (2.90%) and recovered dauers (2.92%). These results suggest that the larvae undergoing dauer development are not genetically different and that there are no transgenerational effects present from being dauers, at least none that perdure for longer than one generation.

**Table 5:** No transgenerational effects of being a dauer or J2A larvae compared to a J2/J3 in terms of offspring produced and likelihood to become dauers in the next generation.

Worm Stage Plated	Offspring after 48h of laying	Dauers after 14 day incubation (%)
J2/J3	78.46 ± 7.25	2.95 ± 0.36
Dauer	73.87 ± 5.31	2.90 ± 0.52
J2A	73.80 ± 4.76	2.92 ± 0.46

There is no difference in production of offspring or dauer formation between any of the starting populations. For each starting population, 10 plates were picked and the experiment was repeated three times. Data shown here is a mean of all samples plus the standard deviation. A students t test and Mann Whitney U were carried out to determine statistical significance between the different treatments, none was found.

### *R*. *diutinus* alters its reproductive output in response to crowding

**Fig 7:**
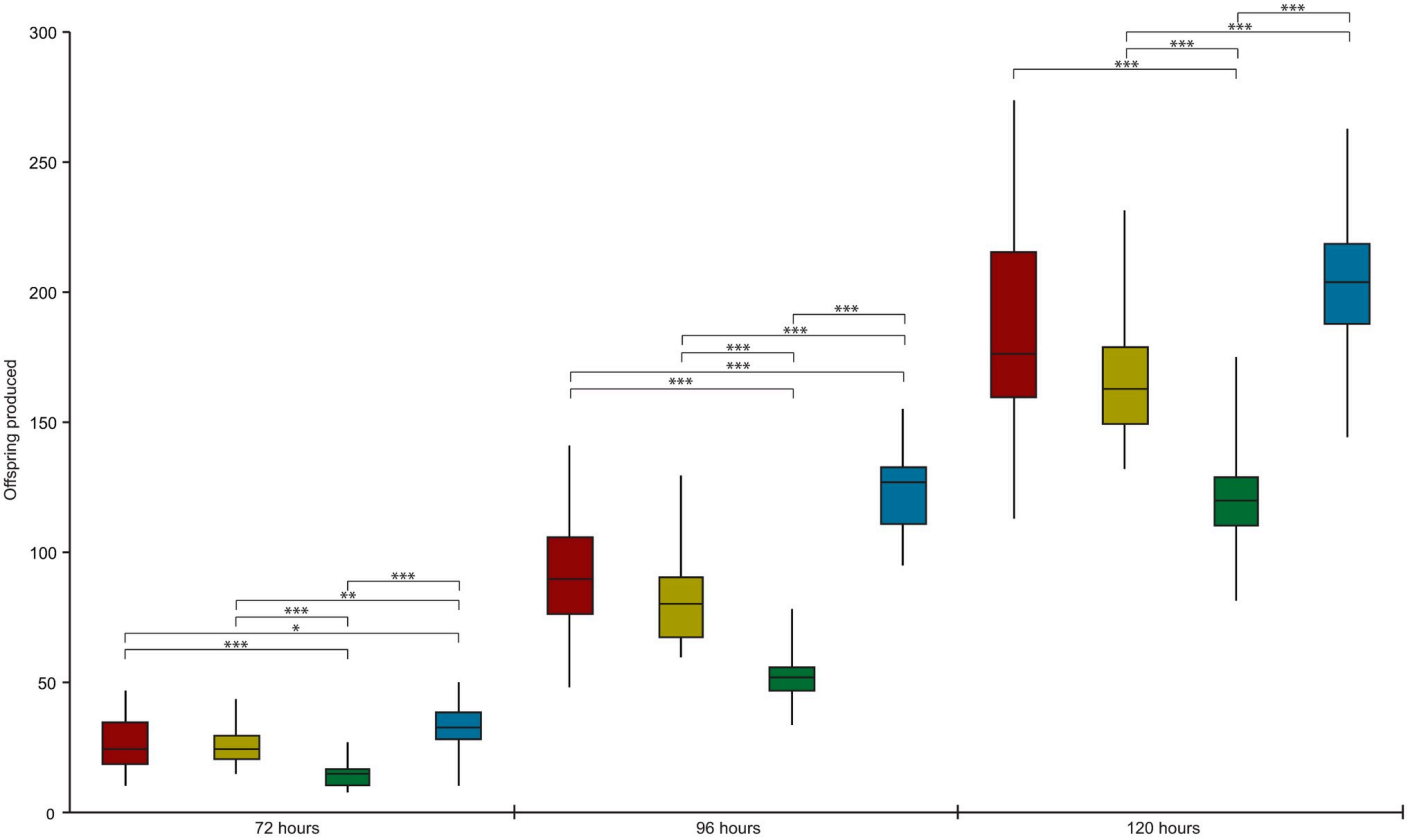
*R*. *diutinus* alters its reproductive output in response to density. *R*. *diutinus* J2s were picked either singularly (red), in groups of 5 (yellow) or 10 (green) onto plates and maintained for 120 hours or picked singularly onto plates and transferred daily to a new plate (blue). Offspring produced after 72, 96 and 120 hours was recorded. ANOVAs were performed between the different samples at each time point. For each density, at least 10 plates were picked, and the experiment was repeated three times. Data is shown as median (line), interquartile range (box) and range of values (whiskers). * indicates a statistically significant value of between 0.05 and 0.01, ** indicates a statistically significant value of between 0.01 and 0.001 and *** indicates a statistically significant value of lower than 0.001.

While there was no difference in fecundity between never starved adults and recovered either J2A or dauer larvae (Table 5), it was noticed during a pilot study that there was a substantial difference in fecundity based upon how many larvae were initially plated. To confirm this, J3 were transferred onto fresh NGM plates with OP50 either singly or in groups of 5 or 10. They were then allowed to develop for 2 days until they started laying embryos. The numbers of embryos laid along with any hatched offspring were then counted for 3 days. For some of the single worm plates, the adult was transferred daily to a new plate. As seen in Fig 7, there is a statistically significant decrease in the number of offspring produced on the first day among the different populations. Whilst there is no statistically significant difference between the single worm and 5 worm plates, there is a strong decrease between 1 worm and 10 worms, with worms maintained in groups of 10 producing nearly half as many eggs on average (14.49) as compared to worms alone on a plates (26.77) (p-value <0.0001). 24 hours later, this pattern of decreased fecundity in response to crowding still persists, with worms kept in groups of 10 producing 52.30 offspring on average compared to 82.29 offspring for groups of 5 and 90.39 worms for single worms (p-values both <0.0001). Most surprisingly however, there is a strong significantly significant decrease in the number of offspring produced between single worms kept on the same plate (90.39) versus those transferred to a new plate daily (123.16) (p-value <0.0001). 24 hours later this pattern remains, with single worms transferred daily having produced an average of 203.48 offspring compared to single worms kept on the same plate (188.10), worms kept in groups of 5 (167.22) and 10 (120.45). This strong correlation between crowding and a reduction in offspring, suggests that *R*. *diutinus* senses its offspring and adjusts its reproductive output based upon how many offspring are already present.

## Discussion

### *Rhabditophanes diutinus* life cycle

*Rhabditophanes diutinus* produces a very low amount (~3% in our experimental settings, Table 5) of dauers in response to starvation. It is unclear as to why dauers in this species have not been previously reported, particularly seeing as dauers were originally reported for other species in this genus over 80 years ago [36]. To our knowledge, this study provides the first example of a fully free-living Clade IV nematode and the closest free-living nematode to any parasite that can be induced to enter and exit a dauer stage under laboratory conditions.

In addition to the production of dauers, *Rhabditophanes diutinus* uses the formation of arrested J2As as a second strategy to survive environmental stress such as lack of food. While other free-living nematodes can also arrest their development at an earlier stage than dauer upon starvation (i.e. *C*. *elegans* [44] or *P*. *pacificus* (R. Sommer, personal communication)), this function is performed by specialised forms of the developmental stage that leaves the egg shell (J1 in *C*. *elegans*, J2 in *P*. *pacificus*) and can therefore develop with yolk as the only food source. In these cases, the survival stages are only physiologically but not morphologically different from their rapidly developing counterparts. In contrast, *R*. *diutinus* forms a physiologically and morphologically different specialized version of J2s, even though J1s are the stage that emerges from the egg shell. In our experiments, J2As survived starvation only moderately longer than J2s. However, J2As were induced by starvation, such that these individuals had been starving prior to the experiment while the J2s were picked from well-fed plates. Therefore, the increase of starvation tolerance was underestimated in our experiments. Nevertheless, one could question if this relatively small benefit would be sufficient for the evolution of a different developmental stage. One could also speculate that the development into J2A does not primarily occur in order to prolong survival after reaching the stage, but because it is easier to enter under starvation conditions. It is imaginable that J2A formation might be less energy consuming than development into a normal J2, for example due to its under-replicated germ line. As such, development to J2A instead of J2 might therefore allow a larva to complete an already initiated J1 to J2 transition upon starvation, thereby avoiding short-term death.

Dauer larvae survive starvation clearly longer than the J2A or J2 larvae but their survival in our experiments was much shorter than was described by [45] for *C*. *elegans* or *P*. *pacificus* dauer larvae, albeit under different conditions. Together with our observation that only a small proportion of *R*. *diutinus* worms formed dauer larvae, as a result of high population density associated with food deprivation, this may suggest that survival of starvation is not the most important purpose of the dauer stage in *R*. *diutinus*. Response to other environmental stresses (i.e. temperature, salinity, osmolarity, oxygen concentration) or dispersal through phoretic associations with other organisms, as was observed in other species of *Rhabditophanes* [36,37] may be more important functions of dauer larva in *R*. *diutinus*. It remains to be tested if such stressors or the presence of putative phoretic partners are more potent inducers of dauer development than starvation or population density. In the closely related facultative parasite *P*. *trichosuri* an iL3-inducing pheromone, as well as an influence of temperature on iL3 development, have already been described [46].

### Potential of *Rhabditophanes diutinus* as a research model

Like other free-living species, *R*. *diutinus* can be easily maintained on standard NGM plates supplemented with OP50, can be frozen following standard protocols for other nematodes [47] and has a high quality genome [25], for which the gene annotations can be improved using the transcriptome data presented in this manuscript. The conservation of function (survival of starvation), morphology, developmental stage (J3), physiology (SDS resistance) and a crucial endocrine regulatory module (DAF-12) strongly suggests that the *R*. *diutinus* dauers are homologous with *Strongyloides* spp. infective larvae and are not an analogous survival stage newly acquired upon reversal from parasitism. Of course, we cannot completely exclude that infective larvae reverted to being dauers, but we think that our results along with [35] make the hypothesis of a reversion from parasitism very unlikely to be true and suggests that the Strongyloididae represent one of multiple independent events of transitioning to a parasitic life style within nematodes, that occurred within the Strongyloidoidea after the split of the families Strongyloididae and Alloionematidae. All species (parasitic, facultative parasitic or non-parasitic) of Strongyloidoidea examined lack almost the same dauer pathway genes from *C*. *elegans*, illustrating that the vast majority of the genomic differences between *C*. *elegans* and *Strongyloides* spp. are due to the enormous phylogenetic distance and not the fact that one taxon is parasitic and forms infective larvae while the other is free-living and makes dauer larvae. The consistent lack of the same insulin signaling pathway genes in Strongyloidoidea together with the recent finding that most of these genes are restricted to the genus *Caenorhabditis* [48] rather points towards recent duplication events in the *Caenorhabditis* lineage than towards a loss in Strongyloididae. While our gene expression analyses are consistent with the hypothesis that some elements of the genetic control of dauers are conserved because the genes are expressed at the right time, the transcriptomic results cannot be interpreted as evidence that these genes do indeed participate in any part of dauer control in *R*. *diutinus*, be it formation, maintenance or exit. For this, functional studies need to be done.

As opposed to comparisons between *Strongyloides* and *C*. *elegans*, it is far likelier that differences in gene content or expression between *Rhabditophanes* and *Strongyloides* are due to the change in lifestyle, although some differences may still be due to phylogeny.

The presence of closely related, cultivable, experimentally tractable parasitic, facultative parasitic and free-living species renders the Strongyloidoidea an excellent example for studying the evolution of parasitism. The dauer hypothesis is an attractive and plausible hypothesis for the evolution of this taxon, which therefore represents a test case for the further study and testing of this hypothesis, that may also apply to several other transitions to parasitism within nematodes [7]. In addition to studying evolutionary parasitism, the Strongyloidoidea are also of interests for studying other fundamental biological processes. For example, it contains two independent transitions to parthenogenetic reproduction, one within the genus *Rhabditophanes* (females of *R*. *diutinus* are parthenogentic [25] while males do exist in other species of *Rhabditophanes* [36,37]) and the second after the split of *Parastrongyloides* (which reproduce sexually in the parasitic generation) and *Strongyloides* (which is parthenogenetic as a parasite) [29].

Finally and most importantly, given *R*. *diutinus*’s phylogenetic proximity to *Strongyloides*, it will be much more similar than *C*. *elegans* is both genomically [25] and with respect to many biological aspects as has previously been shown for vulva development [31] or gonad structure [32]. Therefore *R*. *diutinus* represents an easy to work with, non-parasitic model species for *Strongyloides*, that allows safe experimentation without the need for laboratory animals to study many aspects of these important veterinary and human pathogens.

## Methods

### Species and strain

*Rhabditophanes* sp. KR. 3021 (*diutinus*) was originally isolated in 1994, from a conifer forest floor soil sample by one of us (AR), near the Bamfield Marine Station on the West coast of Vancouver Island, British Columbia. The strain was sent to our lab by Dee Denver (Oregon State University) in July 2014. It has been since maintained in the lab at 15°C on NGM plates supplemented with OP50 bacteria as a food source or as frozen stock (frozen according to [47]). This strain is the same as that in the recent *Strongyloididae* genome paper [25].

### Staging of worms and determination of dauer molts

Worms were picked manually from mixed stage culture plates into 5μl of water on a 4% agarose slide and visualized under a microscope using DIC optics [49]. For the dauer staging, dauers were isolated from 2 week old plates that had been starved of food for at least a week and then visualized directly using DIC microscopy on a Zeiss Imager M2 microscope with a Zeiss Axiocam 506 mono camera. To determine the dauer recovery and number of molts, dauers were picked from the same plates onto fresh NGM plates supplemented with OP50 in groups of 5 and then incubated at 15°C. A plate was removed every 2 hours (upto 44 hours) and the worms on the plate were examined under 40x DIC microscopy to determine stage and changes in development between time points. High magnification images of the different stages was taken using a Zeiss Image Z1 with a Zeiss Axiocam 506 mono camera.

### SDS treatment of dauers and other stages

Old (minimum 2 weeks) plates that had run out of food supply and contained dauers, J2As and a small number of long-living adults were washed off into 1% SDS and incubated gently shaking for 20 mins. Following this, the worms were washed multiple times with water and then recovered on NGM plates with OP50 at 15°C. 1 hour later, plates were examined for surviving larvae and their developmental stage was scored.

### Dauer survival determination

Dauers J2As (from starved plates) and J2/J3s (from well fed plates) were picked in groups of 5 onto NGM plates without any bacteria and incubated for 15 days at 15°C. The plates were checked daily and the number of surviving worms counted. There were 3 biological replicates for each life stage, with each replicate consisting of 5 plates.

### Test for transgenerational effects from dauers

To examine for any transgenerational effects, dauers, J2As and J2/J3s were picked onto fresh NGM plates with a lawn of OP50 and incubated at 15°C for upto 15 days. Plates were checked daily until they began producing offspring. From this day, the original worms were allowed to lay embryos for a further 2 days at which point they were removed. Plates were checked daily to count the number of offspring produced. Once the worms had been removed, the plates were then incubated at 15°C for 10 days after which the amount of dauers and J2A were counted on every plate. Worms were picked onto plates originally in groups of 10, with 10 plates per life stage. This experiment was repeated three times.

### Fecundity determination

To examine fecundity changes, J3 worms were picked either singularly, in batches of 5 or 10 onto fresh NGM plates with a lawn of OP50 and incubated for 48 hours at 15°C to allow development to adults. Following this, plates were examined and total number of offspring (laid eggs and hatched larvae) were counted along with the number of adult worms still alive on the plate. For plates with multiple original larvae, the adults were left on the plate overnight before counting the offspring again. For plates with single worms, the adults were either left on the plate or transferred to a fresh plate and incubated again overnight before counting the offspring. This was repeated once more so that 3 days of offspring production were counted (total of 120h from the original picking of the J3s). At least 10 plates were picked per treatment, with this experiment being repeated three times.

### Bioinformatics analysis of dauer pathway in Strongyloidoidea

To screen for the orthologs of candidate *C*. *elegans* genes in six species of Strongyloidoidea [25], we obtained protein and genome data sets for *C*. *elegans* (WBPS13), *S*. *ratti* (WBPS13), *S*. *stercoralis* (WBPS14), *S*. *venezuelensis* (WBPS14), *S*. *papillosus* (WBPS13), *P*. *trichosuri* (WBPS14), and *Rhabditophanes diutinus* (WBPS14) from WormBase ParaSite [50]. We further selected a broadly sampled set of high quality nematode genomes consisting of *T*. *spiralis* (WBPS13), *B*. *malayi* (WBPS13), and *P*. *pacificus* (El Paco version 2) [40]to be included in the homology and phylogenetic analysis. In case of multiple isoforms for a given gene, the isoform with the longest protein product was chosen as a representative sequence. We then applied a set of complementary approaches to derive the orthology relationships. These included all-against-all blastp searches, orthologous clustering, best-reciprocal hit (BRH) identification, phylogenetic analysis, phylostratigraphy and manual inspection. First, we defined orthologous groups based on a Markov clustering algorithm as implemented in the software orthAgogue which takes the results of all-against-all blastp searches (E-value<0.00001) as input [51]. Protein sequences corresponding to orthologous groups were aligned by the program MUSCLE (version 3.8.31) and phylogenetic trees were computed by RAxML (version 8.2.11, with the PROTGAMMAILG model and fast bootstrapping -f a -N 100) and the resulting trees were manually inspected to test whether their topology roughly corresponded to the species phylogenies [52,53] which is a reliable signature of one-to-one orthologs. In the case that no orthologous sequence for the most robust outgroup species *T*. *spiralis* could be found, we tested if the protein sequences from Strongyloidoidea formed a monophyletic group with regard to all other species. The phylogenetic tree was also useful to disentangle inparalogs from outparalogs if the orthologous cluster contained more than one sequence per species. Second, if a candidate gene from *C*. *elegans* was not part of any orthologous cluster, we screened for BRHs of *C*. *elegans* candidates in all other species and repeated the phylogenetic analysis as described above to identify further one-to-one orthologs. Third, for the remaining candidates we investigated estimates of gene ages as inferred by a recent phylostratigraphic analysis [40,54] to detect recently evolved *C*. *elegans* genes that consequently do not have a homolog in Strongyloidoidea. Notably, many of the insulin related peptides fall under this category. They could be identified by searches with profile hidden Markov Models as implemented by the hmmsearch program of the HMMER package (version 3.0, e-value < 0.001) and extracting hits for the PFAM profile PF03488 (Nematode insulin-related peptide). Even though some level of sequence similarity exists with related sequences in Strongyloidoidea, the amount of sequence divergence prohibits recognition as homologs by BLASTP, which is a commonly used criteria to define novel genes [55]. Finally, for all remaining candidates, we performed phylogenetic analyses based on a set of manually extracted best BLASTP matches in all investigated genomes. For the complete orthology analysis, it was extremely helpful to have multiple genomes of Strongyloidoidea as these serve as independent biological replicates to support gene gains and losses which were usually phylogenetically consistent as they could be explained by a single evolutionary event [48]. A handful of inconsistencies such as secondary losses in individual species could be explained by problems in genome assembly and gene annotation as additional homology searches with the program exonerate (version 2.2.0) [56] could identify at least partial matches with the candidate *C*. *elegans* genes. A complete orthology list including the method using to determine the orthology relationship for each Strongyloidoidea species examined can be found in S2 File.

### Transcriptomic analysis of *R*. *diutinus*

Dauers, J2As, J2/J3 larvae and Adults were isolated and had their RNA extracted using Trizol (Zymo Research) for transcriptome analysis. All stages were picked manually onto bacteria-free NGM plates to reduce excess bacterial contamination. Once a minimum of 200 worms had been transferred, the plates were washed off with H_2_O and the worm pellet concentrated by slow centrifugation (4000g, 2 mins). The worm pellet was then removed in 20μl and transferred to a new RNA free tube, 500μl Trizol added, and frozen instantly in liquid nitrogen. Samples were then disrupted using a motorized pestle until thawed, vortexed for 15 seconds and refrozen in liquid nitrogen. This freeze-thaw cycle was repeated 6 times to ensure complete disruption of the cuticle and homogenization of the sample. Following the final freezing, samples were incubated at 37°C for 5 mins, after which 500μl of Trizol was added and incubated for a further 3 mins. 200μl of Chloroform was added, mixed and incubated for 3 mins and then centrifuged at 12000g for 15 mins. Following this, the aqueous phase was removed and the RNA extracted using RNA Clean and Concentrator-25 Kit (ZymoResearch) as per the manufacturers’ protocol. The RNA was eluted into a final volume of 30μl of RNase/DNase free water and then immediately examined using NanoDrop (PEQLAB Biotechnologie) and Qubit (Invitrogen) to assess its purity and concentration. For all 4 stages of worms, 3 separate biological samples were generated. RNAseq libraries were then prepared using TruSeq RNA Library preparation kit v2 (Illumina) according to the manufacturer’s instructions from 160–1000ng of total RNA in each sample. Between 12 and 15 PCR cycles were used depending upon the original quantity of RNA used. Libraries were quantified by Qubit and Bioanalyzer measurements (Agilent) and normalized to 2.5nM. Samples were then sequenced on a multiplexed lane of an Illumina HiSeq3000 instrument, resulting in 10–20 million 150bp paired end reads for each sample. All sequencing data was submitted to the European Nucleotide Archive under the study accession PRJEB39019. Sequencing adapter sequences were removed using the bcl2fastq software (version 2.18.0.12) with user defined parameter barcode-mismatches set to 1. No additional quality filtering was applied. The resulting sequencing data were aligned against the *Rhabditophanes* genome (WBPS14) with the help of TopHat2 (version 2.0.14, -I 10000 option). Expression levels were estimated by cufflinks (version 2.2.1, default options). Differential expression analysis was done with the cuffdiff software (version 2.2.1, default options). Only genes with a log_2_ fold change either greater than 2 or lower than -2 and a FDR-corrected p-value of less than 0.05 were determined to be significantly differentially expressed. For visualization purposes, expression values in fkpm were transformed into z-scores using the R function “scale”. The z-scores denote the expression difference (in the unit of standard deviations) of a given developmental stage with regard to the average expression level across all stages. Principal component analysis (PCA) was done by the prcomp function (with the scale = T option) of R. Data for the transcriptome profiles of *S*. *papillosus* and *C*. *elegans* were taken from [11,57].

### Dafachronic acid experiments

Dafachronic acid experiments were performed similarly to as described in [13]. OP50 was grown overnight in LB medium and then centrifuged and resuspended in 1/5th volume of 0.9% NaCl. 90μl of resuspended bacteria and either 10μl of 10 or 100μM Δ7 dafachronic acid (diluted in ethanol) or ethanol were combined and spotted on an NGM plate. L3 worms were added to the plate and incubated for 14 days at 15°C after which the plates were examined and all worms staged.

### Statistical analysis and figure generation

Appropriate statistical analysis was carried out using Excel and R with statistical significance determined as being reached once the p-value was below 0.05. The exact statistical test used is noted in the figure or table legends. Microscopy images were resized in Photoshop and then annotated in Illustrator. Figures were generated in Excel, R and Illustrator.

## Supporting information

S1 File Species description and stage description of *Rhabditophanes diutinus*—Morphological descriptions for adults, dauers, J2As and J2s including measurements. (PDF)

S2 File Full data set with which all figures were generated, list of orthologs present within the Strongyloidoidea and full results of the transcriptomic study. (XLSX)

S1 Fig Clustering analysis of transcriptomic biological replicates. (PDF)
