# Supplementary material for: Rhabditophanes diutinus a parthenogenetic clade IV nematode with dauer larvae
Source: PLoS Pathog. 2020 Dec 3;16(12):e1009113. doi: 10.1371/journal.ppat.1009113 (PMC7738172; doi:10.1371/journal.ppat.1009113)
Supplement: S1 File — (PDF) [file ppat.1009113.s001.pdf]

Supplementary File 1: *Rhabditophanes diutinus*, Species Description

Adult: This species consists of only females. Cylindrical body shape, around 900μm long and 60μm wide. Lip regions consists of four sectors, each with a labial sensillum. Narrow buccal cavity with large pharynx consisting of a long narrow cylindrical procorpus, a small rounded metacorpus, a long thin cylindrical isthmus and a large circular well-rounded posterior bulb. Nerve ring sits over isthmus. Grinder appears present within the posterior bulb. Excretory pore present in the isthmus region. Intestine runs length of body, 28μm wide, from directly after the pharynx to the rectum. The intestine is not fixed to the body wall and is wrapped around the gonads. Well- developed sphincter following gonadal loop. Rectum is short and wide, with anus a raised opening in the form of a vertical slit in the cuticle. Vulva located at mid body, with a horizontal slit. Gonads are didelphic and wrap around the intestine, posterior arm is as a result normally hidden by the intestine. The gonad arms extend past the vulva in both directions. Germ cells are arranged in giant nuclei which are easily observable under DIC (10-12μm in size). Following the gonadal loop, undeveloped oocytes are present. These oocytes have a shiny appearance and are rich in cytoplasmic material. Whilst it appears that there is a spermatheca, these cells do not have nuclei. Maturing oocytes pass through them, after which they begin developing into embryos. Embryos are less common in other species and it is rare to see ever more than 2 per gonad arm in development. The exact development stage of the embryo when laid appears inconsistent. Embryos are around 55μm when laid.

Dauer Larvae: Cylindrical body shape, around 450μm long with a large amount of radial constriction (20μm wide at midpoint). Dauers are marked by the presence of a large buccal and intestinal plug consisting of multiple parts. Buccal cavity is narrow and restricted, with a large buccal plug. Further plugs are found in the upper third of the intestine, mid intestine, and lower third of intestine. These plugs appear shiny when viewed under DIC. The rest of the pharynx is constricted, with the posterior bulb appearing more pentagonal in shape than its usual spherical. Gonadal development is limited, consisting of around 10-20 cells, within a small smooth structure. Gut lumen is large and full of bacteria. No vulval development. Anus and rectum appear closed and are much narrower than usual. Cuticle is thicker and striated compared to adult stages.

J2A Larvae: Cylindrical body shape, around 300μm in length, proportionally appears as J2 worm. Pharynx consists of 32% of total body length. Buccal cavity is narrow and open, pharynx fully developed. Intestine appears empty, lumen wall is thinner than other stages. Gonad consists of two to four progenitor cells. All other intracellular organelles appear endocytosed dependent on how long the worm has been in this stage for. Tail is short and triangular. Cuticle is striated. When in this stage for a prolonged period of time, the only identifiable features are the mouth, intestine and germline.

J2 Larvae: Cylindrical body shape, around 350μm in length. Pharynx consists of 28% of total body length. Buccal cavity is open and proportional, around 1.14μm in diameter, pharynx otherwise is fully developed. Intestine appears full of food and the lumen is much narrower (2μm) compared to the intestinal walls (6μm). Gonad consists of 4 cells in early J2, yet the gonad undergoes growth during the J2 stage and by the end often consists of around 20 cells. Rectum and anus appear normal and proportional for the stage. No vulval development present.

Measurement	Adult	Dauer	J2A	J2
**Body Length**	918.50 (836.28-968.77)	460.31 (392.08-512.09)	297.69 (284.88-312.60)	362.32 (329.98-406.43)
**Buccal Cavity Width**	3.29 (2.50-3.93)	n/a	0.95 (0.80-1.14)	1.14 (0.94-1.64)
**Isthmus Width**	10.71 (9.26-12.20)	17.02 (14.93-20.21)	4.61 (3.72-5.02)	5.68 (4.48-6.71)
**Terminal Bulb Width**	29.94 (23.98-34.29)	4.75 (4.28-5.09)	16.87 (15.75-17.75)	13.31 (12.15-14.23)
**Body Width at Proximal Bulb**	37.40 (34.55-42.38)	10.73 (10.00-12.19)	11.63 (11.13-12.51)	19.60 (18.49-20.89)
**Body Width at Grinder**	49.23 (43.49-52.20)	20.71 (18.43-23.27)	19.18 (18.39-20.32)	23.32 (21.65-26.83)
**Pharynx Length**	152.89 (141.38-170.74)	111.77 (103.63-117.51)	94.69 (91.45-97.50)	100.60 (91.84-110.35)
**Pharynx Length of Body Length (%)**	16.65 (15.61-18.55)	24.41 (22.30-29.27)	31.84 (29.50-34.23)	27.84 (25.36-29.59)
**Intestinal Wall Width**	11.58 (8.72-14.57)	4.57 (3.81-5.73)	3.39 (2.27-4.03)	6.52 (5.45-8.63)
**Intestinal Lumen Width**	4.90 (3.29-6.86)	5.42 (4.20-6.90)	3.82 (3.23-4.68)	2.07 (1.32-3.07)
**Ratio of**	0.42 (0.29-0.57)	1.19 (0.91-1.40)	1.17 (1.00-1.61)	0.32 (0.24-0.50)

**Intestinal Lumen to Intestinal Wall**				
**Vulva/Germ cells from anterior**	464.68 (426.18-526.99)	19.81 (17.80-21.92)	123.29 (114.03-137.76)	184.79 (146.50-206.08)
**Vulva/Germ cells from anterior as % of body length**	50.62 (46.20-55.55)	45.65 (40.93-50.22)	41.44 (38.79-46.82)	51.16 (42.87-61.24)
**Body width at Vulva/Germ cells**	60.36 (56.59-64.51)	209.28 (184.59-229.26)	15.83 (14.92-16.99)	20.99 (18.52-26.33)
**Gonad length (anterior tip to loop)**	273.34 (232.99-307.65)	67.35 (57.39-77.54)	12.80 (11.08-14.20)	24.94 (17.94-29.64)
**Tail length (anus to tail tip)**	102.15 (83.23-128.45)	57.27 (51.20-62.03)	51.32 (43.17-56.05)	57.12 (49.47-71.81)

All measurements are in µm unless stated, mean stated and range shown in brackets. As J2A, dauers and J2 do not have a developed vulva, the midpoint of the germ cell block is used in its place. At least 10 randomly selected individuals for each stage were measured.
