# Supplementary figures and images for: Rhabditophanes diutinus a parthenogenetic clade IV nematode with dauer larvae
Source: PLoS Pathog. 2020 Dec 3;16(12):e1009113. doi: 10.1371/journal.ppat.1009113 (PMC7738172; doi:10.1371/journal.ppat.1009113)

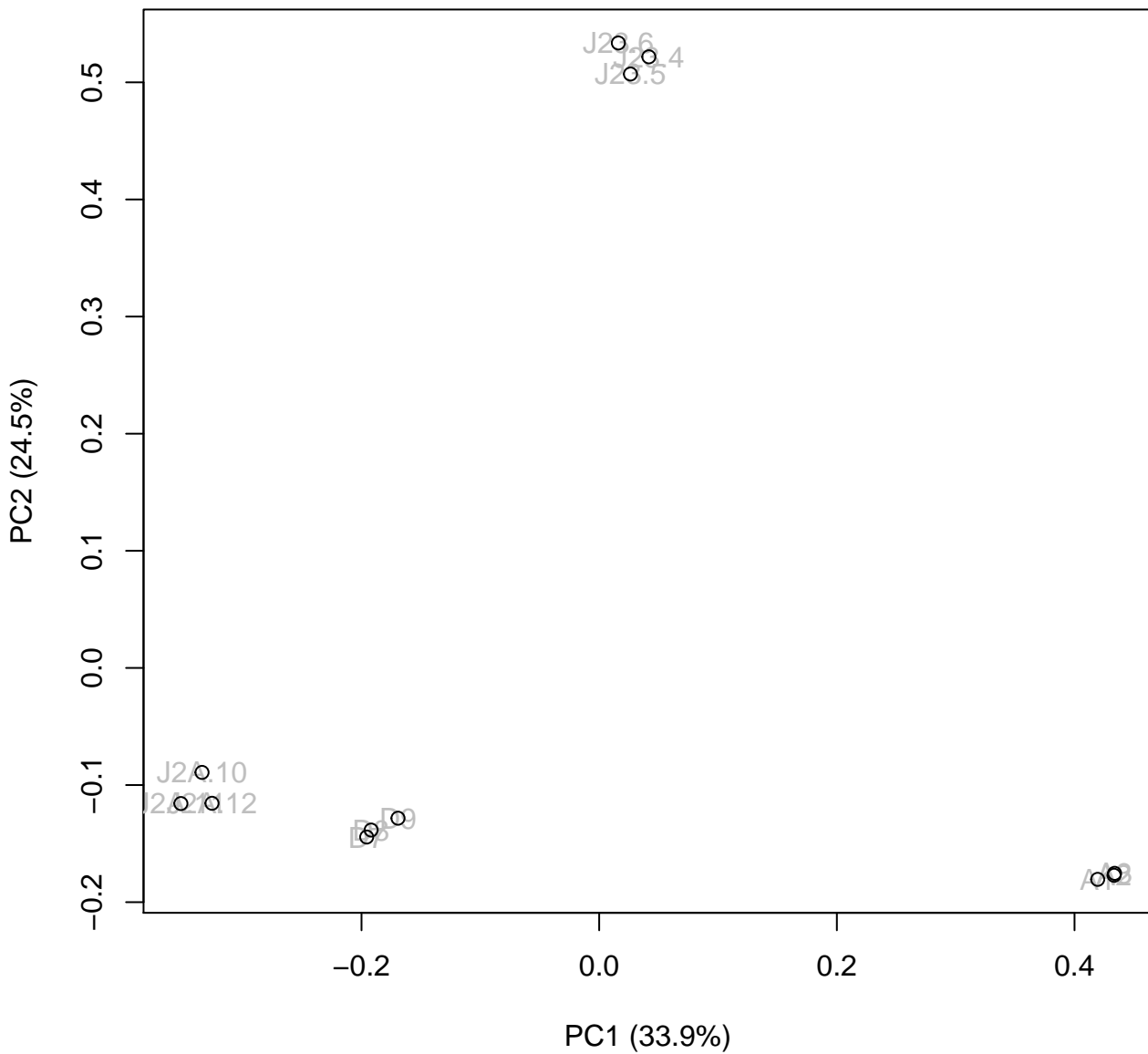

Supplement: S1 Fig — (PDF) [file ppat.1009113.s003.pdf]
